# Supplementary material for: Design and Transition of an Emergency E-Learning Pathology Course for Medical Students—Evaluation of a Novel Course Concept
Source: Eur J Investig Health Psychol Educ. 2023 Jan 9;13(1):112–29. doi: 10.3390/ejihpe13010008 (PMC9858035; doi:10.3390/ejihpe13010008)
Supplement: Supplementary file 1 [file ejihpe-13-00008-s001.zip › ejihpe-2084739-supplementary.pdf]

Supplemental Table S1: Overview of the online courses

| | Number and Format of Teaching units | Duration min. | Duration max. | Number of tasks | Number of questions per task | Types of questions | Average Score |
|---|---|---|---|---|---|---|---|
| **What is pathology?** | 4 Screencasts | 8:23 | 1:11:23 | 4 | 3-3-2-3 | MC / Assignment | 1.47 |
| **Morphological basics** | 9 Screencasts + Introduction | 3:40 | 21:14 | 10 | 1-1-1-2-1-1-1-2-1-2 | MC / Assignment | 1,63 |
| **Artheriosclreosis** | 8 (7a + 7b) Lectures | 4:58 | 16:09 | 7 + PPT slides (homework) | 2-2-2-2-2-2-2 | MC | 1.28 |
| **Inflammation** | 10 Lectures + Introduction | 15:07 | 52:44 | 8 | 1-1-2-2-1-6-2-2 | Drag-and-drop /MC | 1.71 |
| **Immunology** | 3 Lectures | 34:39 | 47:46 | 1 | 3 | MC | 1.56 |
| **General tumor pathology** | 10 Lectures (mit 5 Themen) | 5:47 | 10:09 | 5 + PDF slides (homework) | 1-2-2-1-1 | MC | 1.41 |
| **Immuno-oncology** | 2 Lectures | 42:20 | 45:36 | 1 | 2 | MC | 1.25 |
| **Gynaecological tumor pathology** | 12 Lectures (6 subjects) | 7:14 | 33:39 | 5 + PDF slides (homework) | 9-6-5-3-4 | MC | 1.78 |
| **GI Tumor Pathology—GI** | 6 Lectures | 7:24 | 15:23 | 6 + PDF slides (homework) | 1-1-1-1-1-1 | MC | 1.35 |
| **GIl Tumor Pathology—Pancreas** | 4 Lectures | 7:28 | 27:37 | 3 + PDF slides (homework) | 1-1-1 | MC | 1.41 |
| **Head and neck tumors** | 3 Lectures Powerpoint | - | - | 3 | 1-1-1 | MC | 2.29 |
| **Urological Tumor Pathology I** | 3 Lectures Powerpoint with sound (=screencast) | - | - | none | - | - | 1.94 |
| **General Neuropathology—Stroke** | 5 Screencasts + Welcome (Video) + 1 script (PDF) + 1 case vignette (video) | 3:44 | 6:05 | 5 | 3-3-3-3-3 | MC (+ indication of security in the choice of the answer) | 1.0 |
| **General Neuropathology—Craniocerebral Trauma** | 4 screencasts + 1 script (PDF) + 1 case vignette (video) | 4:10 | 6:01 | 4 | 3-3-3-3 | MC | 1.0 |
| **General Neuropathology—Infections** | 2 screencasts + 1 script (PDF) + 1 case vignette (video) | 5:44 | 5:45 | 2 | 3-3 | MC | 1.0 |
| **General Neuropathology—Brain tumors** | 4 screencasts + 1 script (PDF) + 1 case vignette (video) | 4:52 | 11:00 | 4 | 3-3-3-3 | MC | 1.0 |
| **Special Neuropathology—Maldevelopments and developmental disorders** | 6 screencasts + Welcome (Video) + 1 script (PDF) + 1 slide Take Home Messages | 3:36 | 8:20 | 6 | 1-2-2-1-1-1 | Drag-and-drop / open question with short answer / cloze text selection / MC / assign correct answer | 1.11 |
| **Special Neuropathology—Metabolic disorders** | 5 screencasts + 1 script (PDF) + 1 slide Take Home Messages | 2:04 | 5:42 | 5 | 1-1-1-1-1 | Cloze text selection / drag and drop / put terms in a meaningful order | 1.20 |

| | | | | | | | |
|---|---|---|---|---|---|---|---|
| **Special Neuropathology—Demyelinating diseases** | 7 screencasts + 1 script (PDF) + 1 slide Take Home Messages | 3:14 | 7:17 | 7 | 1-2-1-1-2-1-1 | MC / open question with short answer / gap text selection / assign the correct answer | 1.20 |
| **Special Neuropathology—Neurodegenerative diseases** | 6 screencasts + 1 script (PDF) + 1 slide Take Home Messages | 5:13 | 10:14 | 6 | 1-1-2-1-3-1 | Drag-and-drop / put groups of words in a meaningful order / assign the correct answer / MC | 1.20 |
| **Gastrointestinal Tumor Pathology—Liver** | 2 lectures in PPT format | - | - | 2 | 1-1 | MC | 2,0 |
| **Urological Tumor Pathology II** | 3 lectures | 12:43 | 18:49 | 3 | 1-1-1 | MC | 1.65 |
| **Experimental Pathology** | 3 lectures | 5:07 | 25:09 | 2 | 1-1 | MC | 1.71 |
| **Molecular pathology techniques** | 3 lectures | 12:47 | 23:29 | 3 | 1-1-1 | MC | 1.59 |
| **Other tumors** | 3 lectures | 3:48 | 25:03 | 3 | 1-1-1 | MC | 1.5 |
| **Hematological neoplasias** | 3 lectures in PDF Format | - | - | 3 | 1-1-1 | MC | 2.28 |
| **Skin tumors** | 2 lectures | 12:19 | 25:36 | 2 + PDF slides (homework | 1-1 | MC | 1.44 |
| **Thoracic tumor pathology** | 2 lectures | 16:24 | 22:49 | 1 + PDF slides (homework | 1 | MC | 1.65 |
| **Sarcomas** | 2 lectures in PDF Format | - | - | 2 | 1-1 | MC | 2.12 |
| **Pediatric pathology/developmental disorders** | 2 lectures in PDF Format | - | - | 2 | 1-1 | MC | 1.73 |
| **Death/Autopsy** | 2 lectures | 34:10 | 38:24 | 2 | 1-1 | MC | 1.33 |

Supplemental Table S2: An example of a lesson-planning outline (neuroendocrine tumors vs. neuroendocrine carcinomas) drafted during the planning of the project phase

| | **Topic** | **Duration (minutes)** | **Depth of knowledge** | **Project management** | **Teamwork** | **Scientificity** |
|---|---|---|---|---|---|---|
| Seminar | <u>Introductions:</u><br>- Coach<br>- Participants<br>- Short presentation of the course concept<br>- Determination of learning objectives<br>- Clarification of questions | 15 | | | Teamstart | |
| | <u>Review of DigiPath content as it relates to the topic:</u><br>- Questions to the panel concerning basic morphology and general mechanisms of tumorigenesis/metastasis.<br>Coach takes the role of a moderator here, but intervenes as an expert when needed | 30 | Repetition | | | |
| | *- Systematics of neuroendocrine neoplasms. NET vs. NEC* | 15 | Application | | | |
| | <u>Discussion of the content: What are the issues?</u> | 15 | | | | Problem identification |

| | | | | | | |
|---|---|---|---|---|---|---|
| | - NET: biomarkers, therapy/prognosis<br>- NEC: biomarkers, therapy/prognosis<br>- NET vs. NEC: borderline cases<br>*- Distribution of tasks/representations (groups of 2)*<br><br>Preparation of papers for 2nd seminar:<br>- Where to find information?<br>- PubMed | 15 | | Project plan/goals | | Information collection Where? How? What? |
| Homework | Preparation of presentations for 2nd seminar | | | | | |
| Seminar | Presentation and discussion of the presentations:<br>- Presentation 1<br>- Presentation 2<br>- Presentation 3<br>- Presentation 4<br>Determination of group topic (poster topic):<br>- Summary discussion<br>- -Identify and name problem<br><br>*- What additional information do we need?*<br>*- Quality characteristics of scientific information*<br>*- Designation of a milestone paper by the coach* | 60<br>25<br>5 | Consolidation | Planning refinement | Discussion<br>Decision | Presentation discussion<br>Literature research |
| Homework | Working through the milestone paper | | | | | |
| Seminar | Journal Club of the Milestone Paper:<br>- How does a journal club work?<br>- Presentation by the group<br>- Coach presents key fig., etc.<br>- Discussion (weaknesses/strengths/information).<br>Poster design:<br>- Basics (how do you make a poster?).<br>- What content/statements should be included in our poster?<br>- Distribution of tasks<br>Formation of two groups of 4, each to select another paper (relevant for poster/presentation in Journal Club), e.g., BreakOut in Zoom | 45<br>30<br>15 | | Decision regarding allocation of tasks | | Scientific poster |
| Homework | Select and work through the paper | | | | | |
| Seminar | Journal Club of the 2 other papers:<br>- Presentation by group<br>- Discussion<br>- What should be on the poster?<br>Poster design | 45<br>45 | | | Collaborative preparation | Critical evaluation of external scientific data |
| Homework | Delivery of the poster to the course instructor | | | | | |
| Seminar | Discussion of own poster; Discussion of 3 other posters; Reflection | | | | How was it? | Scientific discourse |

Supplemental Table S3: Overall results of long survey

| Results Summer Semester | 2020, N=23 | Missing | Results Winter Semester 2020/2021 N= 27 | Missing |
|---|---|---|---|---|
| **Demography** | | | | |

| | | | | | |
|---|---|---|---|---|---|
| **Age** | | | | | |
| <25y | 21 (95.5%) | | <25y | 19 (70.4%) | |
| 25-30y | 1 (4.5%) | 1 | 25-30y | 8 (29.6%) | 0 |
| >30y | 0 (0.0%) | | >30y | 0 (0.0%) | |
| Gender | | | | | |
| Male | 6 (26.1%) | 0 | Male | 5 (18.5%) | 0 |
| Female | 17 (73.9%) | | Female | 22 (81.5%) | |
| Asynchronous Phase<br>Course units<br>Please rate the course section "What is pathology?" in school grades (1-6) | | | | | |
| | 1.47 (0.61) | 4 | | 1.92 (0.56) | 1 |
| Your remarks about the course section “What is pathology?" | | | | | |
| | *Free-text comments* | | | | |
| Please rate the course section "Morphological basics" in school grades (1-6) | | | | | |
| | 1.63 (1.17) | 4 | | 1.63 (0.65) | 3 |
| Your remarks about the course section "Morphological basics" | | | | | |
| | *Free-text comments* | | | | |
| Please rate the course section "Atherosclerosis" in school grades (1-6) | | | | | |
| | 1.28 (0.58) | 5 | | 1.76 (0.66) | 2 |
| Your remarks about the course section "Atherosclerosis" | | | | | |
| | *Free-text comments* | | | | |
| Please rate the course section "Inflammation" in school grades (1-6) | | | | | |
| | 1.71 (0.92) | 6 | | 2.00 (1.02) | 3 |
| Your remarks about the course section "Inflammation" | | | | | |
| | *Free-text comments* | | | | |
| Please rate the course section "Immunology" in school grades (1-6) | | | | | |
| | 1.56 (0.92) | 5 | | 2.0 (0.71) | 6 |
| Your remarks about the course section "Immunology" | | | | | |
| | *Free-text comments* | | | | |
| Please rate the course section "General tumor pathology" in school grades (1-6) | | | | | |
| | 1.41 (0.51) | 6 | | 1.88 (0.90) | 3 |
| Your remarks about the course section "General tumor pathology" | | | | | |
| | *Free-text comments* | | | | |
| Please rate the course section "Immuno-oncology" in school grades (1-6) | | | | | |
| | 1.25 (0.45) | 7 | | 2.00 (1.02) | 5 |
| Your remarks about the course section "Immuno-oncology" | | | | | |
| | *Free-text comments* | | | | |
| Please rate the course section " Gynecological tumor pathology " in school grades (1-6) | | | | | |
| | 1.78 (0.94) | 5 | | 3.25 (1.5) | 3 |
| Your remarks about the course section " Gynecological tumor pathology " | | | | | |
| | *Free-text comments* | | | | |
| Please rate the course section " GI Tumor Pathology - GI " in school grades (1-6) | | | | | |
| | 1.35 (0.49) | 6 | | 2.29 (0.85) | 6 |
| Your remarks about the course section " GI Tumor Pathology - GI " | | | | | |
| | *Free-text comments* | | | | |
| Please rate the course section " GI Tumor Pathology - Pancreas " in school grades (1-6) | | | | | |
| | 1.41 (0.62) | 6 | | 2.15 (0.75) | 7 |
| Your remarks about the course section " GI Tumor Pathology - Pancreas " | | | | | |
| | *Free-text comments* | | | | |
| Please rate the course section " Head and neck tumors " in school grades (1-6) | | | | | |
| | 2.29 (1.50) | 6 | | 2.65 (1.50) | 7 |
| Your remarks about the course section " Head and neck tumors " | | | | | |
| | *Free-text comments* | | | | |
| Please rate the course section " Urological Tumor Pathology I " in school grades (1-6) | | | | | |
| | 1.94 (1.00) | 7 | | 2.41 (1.01) | 5 |
| Your remarks about the course section " Urological Tumor Pathology I " | | | | | |
| | *Free-text comments* | | | | |
| Please rate the course section " General Neuropathology - Stroke " in school grades (1-6) | | | | | |
| | 1.00 (0.00) | 4 | | 1.24 (0.52) | 2 |
| Your remarks about the course section " General Neuropathology - Stroke " | | | | | |
| | *Free-text comments* | | | | |
| Please rate the course section " General Neuropathology - Craniocerebral Trauma " in school grades (1-6) | | | | | |
| | 1.00 (0.00) | 4 | | 1.17 (0.38) | 3 |
| Your remarks about the course section “General Neuropathology - Craniocerebral Trauma " | | | | | |
| | *Free-text comments* | | | | |
| Please rate the course section " General Neuropathology - Infections " in school grades (1-6) | | | | | |

| | | | |
|---|---|---|---|
| 1.00 (0.00) | 4 | 1.25 (0.53) | 3 |
| Your remarks about the course section " General Neuropathology - Infections " | | | |
| *Free-text comments* | | | |
| Please rate the course section " General Neuropathology - Brain Tumors " in school grades (1-6) | | | |
| 1.00 (0.00) | 4 | 1.21 (0.51) | 3 |
| Your remarks about the course section " General Neuropathology - Brain Tumors " | | | |
| *Free-text comments* | | | |
| Please rate the course section " Special Neuropathology - Maldevelopments & Developmental Disorders " in school grades (1-6) | | | |
| 1.11 (0.32) | 5 | 1.33 (0.57) | 3 |
| Your remarks about the course section " Special Neuropathology - Maldevelopments & Developmental Disorders " | | | |
| *Free-text comments* | | | |
| Please rate the course section " Special Neuropathology - Metabolic Disorders " in school grades (1-6) | | | |
| 1.20 (0.41) | 3 | 1.22 (0.52) | 4 |
| Your remarks about the course section " Special Neuropathology - Metabolic Disorders " | | | |
| *Free-text comments* | | | |
| Please rate the course section " Special Neuropathology - Demyelinating Diseases " in school grades (1-6) | | | |
| 1.20 (0.41) | 3 | 1.22 (0.52) | 4 |
| Your remarks about the course section " Special Neuropathology - Demyelinating Diseases " | | | |
| *Free-text comments* | | | |
| Please rate the course section " Special Neuropathology - Neurodegenerative Diseases " in school grades (1-6) | | | |
| 1.20 (0.41) | 3 | 1.21 (0.51) | 3 |
| Your remarks about the course section " Special Neuropathology - Neurodegenerative Diseases " | | | |
| *Free-text comments* | | | |
| Please rate the course section " Molecular pathology techniques " in school grades (1-6) | | | |
| 1.59 (0.71) | 6 | 2.26 (0.99) | 8 |
| Your remarks about the course section " Molecular pathology techniques " | | | |
| *Free-text comments* | | | |
| Please rate the course section " Experimental Pathology " in school grades (1-6) | | | |
| 1.71 (0.77) | 6 | 2.61 (0.98) | 9 |
| Your remarks about the course section " Experimental Pathology " | | | |
| *Free-text comments* | | | |
| Please rate the course section " Gastrointestinal Tumor Pathology - Liver " in school grades (1-6) | | | |
| 2.00 (1.09) | 5 | 2.45 (1.45) | 7 |
| Your remarks about the course section " Gastrointestinal Tumor Pathology - Liver " | | | |
| *Free-text comments* | | | |
| Please rate the course section " Urological Tumor Pathology II " in school grades (1-6) | | | |
| 1.65 (0.86) | 6 | 2.39 (1.24) | 9 |
| Your remarks about the course section " Urological Tumor Pathology II " | | | |
| *Free-text comments* | | | |
| Please rate the course section " Other tumors " in school grades (1-6) | | | |
| 1.50 (0.52) | 7 | 2.33 (1.03) | 9 |
| Your remarks about the course section " Other tumors " | | | |
| *Free-text comments* | | | |
| Please rate the course section " Hematological neoplasias " in school grades (1-6) | | | |
| 2.28 (1.53) | 5 | 2.15 (0.99) | 7 |
| Your remarks about the course section " Hematological neoplasias " | | | |
| *Free-text comments* | | | |
| Please rate the course section " Thoracic tumor pathology " in school grades (1-6) | | | |
| 1.65 (0.61) | 6 | 2.30 (0.92) | 7 |
| Your remarks about the course section " Thoracic tumor pathology " | | | |
| *Free-text comments* | | | |
| Please rate the course section " skin tumors " in school grades (1-6) | | | |
| 1.44 (0.63) | 7 | 2.37 (1.01) | 8 |
| Your remarks about the course section " skin tumors " | | | |
| *Free-text comments* | | | |
| Please rate the course section " Sarcomas " in school grades (1-6) | | | |
| 2.12 (1.32) | 6 | 2.32 (1.16) | 8 |
| Your remarks about the course section " Sarcomas " | | | |
| *Free-text comments* | | | |
| Please rate the course section " Pediatric pathology/development disorders " in school grades (1-6) | | | |
| 1.73 (1.22) | 8 | 2.35 (0.88) | 7 |
| Your remarks about the course section " Pediatric pathology/development disorders " | | | |
| *Free-text comments* | | | |
| Please rate the course section " Death/Autopsy " in school grades (1-6) | | | |

| | | | |
|---|---|---|---|
| 1.33 (0.62) | 8 | 2.17 (0.72) | 15 |
| Your remarks about the course section " Death/Autopsy " | | | |
| *Free-text comments* | | | |
| Course usability<br>I find it easy to use the AllPathOn course. (1= do not agree at all, 5 = fully agree). | | | |
| 4.77 (0.69) | 1 | 4.38 (0.88) | 3 |
| The structure of the AllPathOn course is clear. (1= do not agree at all, 5 = fully agree). | | | |
| 4.64 (0.90) | 1 | 4.52 (0.73) | 4 |
| Whenever I make a mistake while using this system, the system makes it easy for me to get back to where I wanted to go. (1= do not agree at all, 5 = fully agree). | | | |
| 4.59 (0.85) | 1 | 4.05 (1.13) | 5 |
| No problems were encountered when using the AllPathOn course. (1= do not agree at all, 5 = fully agree). | | | |
| 3.82 (1.05) | 1 | 3.78 (1.72) | 0 |
| If any problems have occurred during use, please describe them briefly. | | | |
| *Free-text comments* | | | |
| Course format<br>I missed the direct interaction in class with the lecturers for a better understanding of the course. (1= do not agree at all, 5 = fully agree). | | | |
| 2.81 (1.44) | 2 | 3.04 (1.34) | 0 |
| The screencasts (PowerPoint slides with voice-over) were a suitable format. (1= do not agree at all, 5 = fully agree). | | | |
| 4.55 (0.74) | 1 | 4.22 (1.09) | 0 |
| The lecture recordings were a suitable format. (1= do not agree at all, 5 = fully agree). | | | |
| 3.79 (1.18) | 4 | 4.13 (1.23) | 3 |
| The animated videos were a suitable format. (1= do not agree at all, 5 = fully agree). | | | |
| 4.63 (0.60) | 4 | 4.63 (0.84) | 0 |
| Course conception and structure<br>The learning objectives are presented in a comprehensible manner. (1= do not agree at all, 5 = fully agree). | | | |
| 4.14 (1.20) | 2 | 3.26 (1.20) | 0 |
| The learning content follows a common thread and builds on itself well. (1= do not agree at all, 5 = fully agree). | | | |
| 4.14 (0.96) | 2 | 3.15 (1.03) | 0 |
| Course content<br>The information provided in the AllPathOn course are clear. (1= do not agree at all, 5 = fully agree). | | | |
| 4.24 (1.04) | 2 | 3.60 (0.91) | 2 |
| I was able to clarify my remaining questions. (1= do not agree at all, 5 = fully agree). | | | |
| 4.08 (1.08) | 11 | 3.67 (1.37) | 9 |
| How did you resolve the remaining questions? | | | |
| E-Mail 2 (15,4%)<br>Telephone 1 (0.0%)<br>Internet 8 (61.5%)<br>Books 4 (30.8%)<br>Fellow Students 5 (38.5%)<br>Other 2 (15.4%) | 10 | E-Mail 1 (4.5%)<br>Telephone 0 (0.0%)<br>Internet 6 (27.3%)<br>Books 5 (22.7%)<br>Fellow students 7 (31.8%)<br>Other 2 (9.0%) | 5 |
| The online activities in this course were associated with clear tasks and objectives. (1= do not agree at all, 5 = fully agree). Asked in summer semester only. | | | |
| 4.21 (0.86) | 4 | N/A | |
| I had difficulty answering the questions asked. (1= do not agree at all, 5 = fully agree). Asked in summer semester only. | | | |
| 1.74 (0.99) | 4 | N/A | |
| I did not understand the following topics correctly: | | | |
| *Free-text comments* | | | |
| Requirement and demand:<br>The amount of time required was… | | | |
| Far too high 1 (4.5%)<br>Too high 1 (4.5%)<br>Appropriate 19 (86.4%)<br>Too low 1 (4.5%)<br>Far too low 0 (0.0%) | 1 | Far too high 2 (7.4%)<br>Too high 13 (48.1%)<br>Appropriate 12 (44.4%)<br>Too low 0 (0.0%)<br>Far too low 0 (0.0%) | 0 |
| The number of teaching topics was… | | | |
| Far too high 0 (0.0%)<br>Too high 4 (19.0%)<br>Appropriate Too 17 (80.7%)<br>low 0 (0.0%)<br>Far too low 0 (0.0%) | 2 | Far too high 9 (33.3%)<br>Too high 12 (44.4%)<br>Appropriate 6 (22.2%)<br>Too low 0 (0.0%)<br>Far too low 0 (0.0%) | 0 |
| How much time did you spend completing the course online per week? | | | |

| | | | | | |
|---|---|---|---|---|---|
| <1h | 1 (4.5%) | 1 | <1h | 0 (0.0%) | 1 |
| 1-5h | 14 (63.6%) | | 1-5h | 3 (11.5%) | |
| 6-10h | 6 (27.3%) | | 6-10h | 6 (23.1%) | |
| 11-15h | 1 (4.5%) | | 11-15h | 8 (30.8%) | |
| 16-20h | 0 (0.0%) | | 16-20h | 6 (23.1%) | |
| >20h | 0 (0.0%) | | >20h | 3 (11.5%) | |
| How much time did you spend doing additional research on the topics offered in the course? | | | | | |
| <1h | 15 (71.4%) | 2 | <1h | 6 (24.0%) | 2 |
| 1-5h | 5 (23.8%) | | 1-5h | 14 (56.0%) | |
| 6-10h | 0 (0.0%) | | 6-10h | 3 (12.0%) | |
| 11-15h | 1 (4.8%) | | 11-15h | 0 (0.0%) | |
| 16-20h | 0 (0.0%) | | 16-20h | 1 (4.0%) | |
| >20h | 0 (0.0%) | | >20h | 1 (4.0%) | |
| Supervision by the lecturers<br>If found that the lecturers were easily contactable. (1= do not agree at all, 5 = fully agree). | | | | | |
| | 4.83 (0.41) | 17 | | 4.00 (1.25) | 12 |
| I was able to clarify all the questions. (1= do not agree at all, 5 = fully agree). | | | | | |
| | 4.67 (0.71) | 14 | | 3.92 (1.44) | 14 |
| The weekly Q&A session has contributed to a better understanding of the content. (1= do not agree at all, 5 = fully agree). (Asked in summer semester only.) | | | | | |
| | 3.14 (1.86) | 16 | | N/A | |
| Learning success<br>I understood the course content. (1= do not agree at all, 5 = fully agree). | | | | | |
| | 3.95 (1.24) | 2 | | 3.16 (1.11) | 2 |
| I feel confident in handling the course content. (1= do not agree at all, 5 = fully agree). | | | | | |
| | 3.30 (1.38) | 3 | | 2.52 (1.05) | 2 |
| Overall Satisfaction<br>I enjoyed attending the course. (1= do not agree at all, 5 = fully agree). | | | | | |
| | 3.59 (1.02) | 0 | | 3.23 (1.31) | 1 |
| I am motivated to continue to explore pathology topics in my studies. (1= do not agree at all, 5 = fully agree). | | | | | |
| | 4.04 (0.88) | 0 | | 3.15 (1.16) | 1 |
| How would you rate the AllPathOn course in general? (1= worst, 5= best) | | | | | |
| | 4.32 (1.21) | 1 | | 3.67 (1.13) | 3 |
| Were there any course days that you felt should be improved, why? | | | | | |
| | *Free-text comments* | | | | |
| Were there any course days that you sectionicularly enjoyed, why? | | | | | |
| | *Free-text comments* | | | | |
| What did you sectionicularly like in general? | | | | | |
| | *Free-text comments* | | | | |
| In your view, what could be improved about the course in general? | | | | | |
| | *Free-text comments* | | | | |
| Flipped Classroom Phase<br>Requirements<br>The task was completely clear to me. (1= do not agree at all, 5 = fully agree). | | | | | |
| | N/A | | | 3.82 (0.96) | 0 |
| I found the group size to be appropriate. (1= do not agree at all, 5 = fully agree). | | | | | |
| | N/A | | | 4.81 (0.49) | 1 |
| How much time did you spend on preparation and post-work during the group work phase? | | | | | |
| | N/A | | <1h | 0 (0.0%) | 0 |
| | | | 1-5h | 7 (25.9%) | |
| | | | 6-10h | 8 (29.6%) | |
| | | | 11-15h | 8 (29.6%) | |
| | | | 16-20h | 3 (11.1%) | |
| | | | >20h | 1 (3.7%) | |
| I found the amount of work to be reasonable. (1= do not agree at all, 5 = fully agree). | | | | | |
| | N/A | | | 4.56 (0.70) | 0 |
| Group atmosphere<br>There was a good working atmosphere in the group. (1= do not agree at all, 5 = fully agree). | | | | | |
| | N/A | | | 4.74 (0.71) | 0 |
| The contact ban in force at the time of the group work phase has affected the cooperation. (1= do not agree at all, 5 = fully agree). | | | | | |
| | N/A | | | 2.67 (1.33) | 0 |
| The pandemic-related contact ban made communication difficult. (1= do not agree at all, 5 = fully agree). | | | | | |
| | N/A | | | 2.63 (1.39) | 0 |
| Supervision by lecturers<br>How satisfied were you with the support you received from the lecturer? (1= worst, 5 = best) | | | | | |

| | | | |
|---|---|---|---|
| N/A | | 4.89 (0.32) | 0 |
| The lecturer was available to answer questions and provide further assistance as needed. (1= do not agree at all, 5 = fully agree). | | | |
| N/A | | 5.00 (0.00) | 0 |
| The lecturer responded sufficiently to questions and suggestions from the students. (1= do not agree at all, 5 = fully agree). | | | |
| N/A | | 5.00 (0.00) | 0 |
| The lecturer formulated criticism in a fair and constructive way. (1= do not agree at all, 5 = fully agree). | | | |
| N/A | | 4.96 (0.19) | 0 |
| Learning gain<br>How would you rate your learning gains in terms of your ability to work scientifically? (1= no gain, 5 = very high) | | | |
| N/A | | 4.15 (0.97) | 0 |
| How would you rate your learning gains in terms of your ability to search relevant scientific literature? (1= no gain, 5 = very high) | | | |
| N/A | | 4.26 (0.81) | 0 |
| How would you rate your learning gain in being able to critically reflect on scientific content? (1= no gain, 5 = very high) | | | |
| N/A | | 4.37 (0.74) | 0 |
| How would you rate your learning gain in being able to work in a team? (1= no gain, 5 = very high) | | | |
| N/A | | 3.52 (0.94) | 0 |
| How would you rate your learning gain in terms of planning and implementing science projects? (1= no gain, 5 = very high) | | | |
| N/A | | 3.82 (1.00) | 0 |
| How would you rate your learning gain in terms of your ability to develop scientific posters? (1= no gain, 5 = very high) | | | |
| N/A | | 4.41 (0.57) | 0 |
| The pandemic contact ban affected my learning gain. (1= do not agree at all, 5 = fully agree). | | | |
| N/A | | 2.63 (1.47) | 0 |
| Overall results of the Flipped Classroom Phase<br>How satisfied were you with the result of your group work (school grades) | | | |
| N/A | | 1.30 (0.47) | 0 |
| The pandemic-related ban on contact affected the outcome of group work. (1= do not agree at all, 5 = fully agree). | | | |
| N/A | | 2.26 (1.26) | 0 |
| The group work increased my interest in pathology. (1= do not agree at all, 5 = fully agree). | | | |
| N/A | | 4.04 (1.02) | 0 |
| The group work phase increased my interest in scientific work. (1= do not agree at all, 5 = fully agree). | | | |
| N/A | | 3.93 (1.14) | 0 |
| How would you rate the group phase overall? (school grades) | | | |
| N/A | | 1.44 (0.51) | 0 |
| What do you find sectionicularly successful with regard to the group phase? | | | |
| *Free-text comments* | | | |
| What suggestions do you have for improvement? | | | |
| *Free-text comments* | | | |

Supplemental Table S4: Overall results of short survey (section of general semester evaluation).

| **Results Summer Semester 2020, N=29** | | Missing | Results Winter Semester 2020/2021 N= 68 | | Missing |
|---|---|---|---|---|---|
| **Please grade the course with a school grade** | | | | | |
| **1.56 (0.85)** | | 2 | 2.32 (1.16) | | 0 |
| The content of the course was presented in a comprehensible way. | | | | | |
| 1.30 (0.46) | | 2 | 2.31 (1.11) | | 0 |
| How much time did you spend working on the course overall? | | | | | |
| <5h | 0 (0.0%) | | <5h | 3 (4.4%) | |
| 5-9h | 1 (3.6%) | | 5-9h | 8 (11.8%) | |
| 10-14h | 3 (10.7%) | 1 | 10-14h | 6 (8.8%) | 0 |
| 15-25h | 4 (14.3%) | | 15-25h | 15 (22.1%) | |
| >25h | 20 (71.4%) | | >25h | 36 (52.9%) | |
| The scope of the course was appropriate. | | | | | |

| | | | |
|---|---|---|---|
| 1.57 (0.68) | 1 | 2.49 (1.29) | 1 |
| Do you have any further remarks? | | | |
| *Free-text comments* | | | |
